# Prediction of Recurrence of Atrial Fibrillation Post-ablation Based on Atrial Fibrosis Seen on Late Gadolinium Enhancement MRI: A Meta-analysis

**DOI:** 10.2174/1573403X19666221205100148

**Published:** 2023-03-22

**Authors:** Manjari Rani Regmi, Mukul Bhattarai, Priyanka Parajuli, Albert Botchway, Nitin Tandan, Jumana Abdelkarim, Mohamed Labedi

**Affiliations:** 1 Division of Cardiology, Southern Illinois University School of Medicine, Springfield, Ill, USA;; 2 Columbia University Division of Cardiology, Mount Sinai Medical Center, Miami Beach, FL, USA;; 3 Department of Internal Medicine, Southern Illinois University School of Medicine, Springfield, Ill, USA;; 4 Division of Cardiology, Ochsner Clinic Foundation, New Orleans, LA, USA;; 5 Division of Endocrinology, Southern Illinois University School of Medicine, Springfield, Ill, USA

**Keywords:** AF ablation, LGE, MRI, left atrium, fibrosis, recurrence

## Abstract

**Objectives:**

This meta-analysis aims to investigate the recurrence of atrial fibrillation (AF) post-ablation based on the various stages of fibrosis seen in the late gadolinium enhancement magnetic resonance imaging (LGE-MRI).

**Methods:**

Electronic databases were searched using specific terms and identified nine studies that met the inclusion criteria. A total of 1,787 patients underwent LGE-MRI to assess atrial fibrosis before catheter ablation for AF. We performed three analyses: first, we compared stage IV *versus* stage I (reference group). The second set examined the combined stages III and IV *versus* stages I and II (reference group). The third set compared stage IV *versus* combined stages I, II, and III. The metanalysis relied on a random-effects model to pool the odds ratios (OR) and 95% confidence intervals (CI) using the DerSimonian and Laird method. The data was analyzed using StatsDirect software in England.

**Results:**

The study showed a higher rate of AF recurrence after ablation in stage IV atrial fibrosis than in stage I (OR, 9.54; 95% CI, 3.81 to 28.89; *P*<00001). Also, in patients with combined stages III & IV of atrial fibrosis, AF recurrence was significantly higher after ablation than in stages I & II groups (OR, 2.37; 95% CI, 1.61 to 3.50; *P*<00001). Similarly, compared to combined stages I, II, and III, patients with stage IV have higher odds of recurrence post-ablation (OR, 4.24; 95% CI, 2.39- 7.52, *P* < 0.001).

**Conclusion:**

This metanalysis demonstrates the strong association between left atrial fibrosis in LGE-MRI and AF post-ablation recurrence. The finding of this study will further assist clinicians in predicting the recurrence rate of AF based on the amount of fibrosis and tailor therapeutic decisions for further management.

## 1 INTRODUCTION

Atrial fibrillation (AF) is one of the most common arrhythmias, which increases the risk of stroke and health care costs and reduces the quality of life and cardiac performance [1]. In 2019, more than 33 million people worldwide were estimated to have AF [2]. One pathophysiology behind AF is atrial fibrosis and loss of atrial muscle mass [3]. Treatment of AF depends upon the individual patient. The treatment options are rate control, rhythm control, electric cardioversion, or percutaneous catheter ablation [3]. Catheter ablation is performed for AF, which is refractory to antiarrhythmic drugs and has become an essential modality of treatment [4]. However, despite emerging technologies in catheter ablation, persistent AF's success rate is still 45-50% in some studies [5]. It is an invasive procedure with potential major complications like stroke, atrio-esophageal fistula, and cardiac tamponade, so predictors for AF ablation are vital to tailor the treatment approach [6]. The degree of left atrial fibrosis has been identified as one of the independent predictors of the recurrence of AF post-ablation [6, 7]. Hence, to quantify the degree of left atrial fibrosis, there-have been studies performed that used late gadolinium enhancement magnetic resonance imaging (LGE-MRI) or delayed enhancement magnetic resonance imaging (DE-MRI) [6, 7]. LGE-MRI has been known to predict the recurrence of AF post-ablation in observational studies [7-15]. However, these individual studies have fewer sample sizes and are single-center studies, except for the study by Marrouche *et al.* [7]. In this paper, we have systematically combined the studies that used LGE-MRI to quantify atrial fibrosis based on the Utah stages of fibrosis before ablation to predict AF's recurrence.

## 2 METHODS

### 2.1 Study Selection

**Fig. (1):**
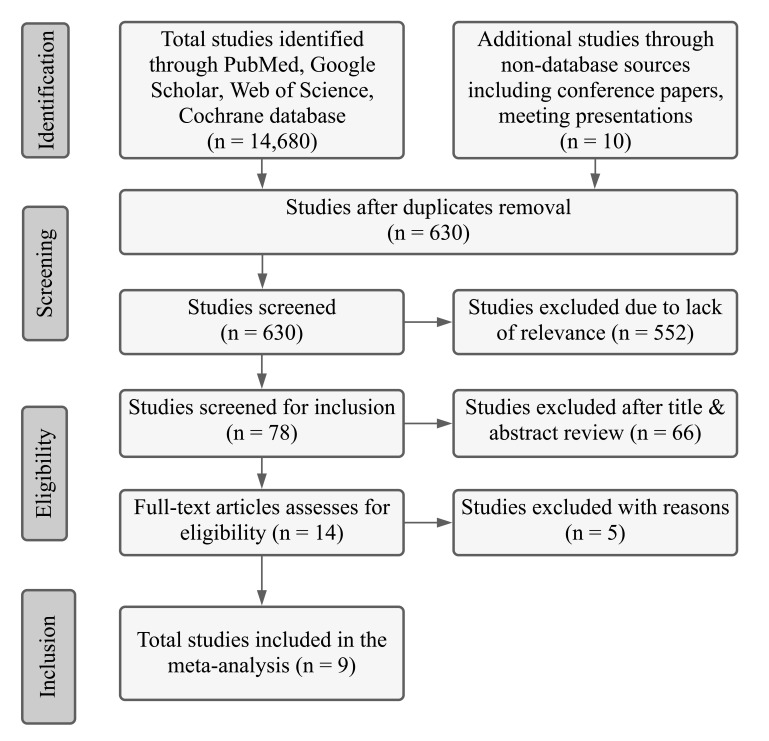
PRISMA flow diagram demonstrating study search strategy used to collect studies for the metanalysis.

We followed the PRISMA (Preferred Reporting Items for Systematic Reviews and Meta-Analyses) flow diagram and provided the search strategy to obtain all eligible studies (Fig. **1**). Two authors (MRR and MB) independently searched PubMed, Google Scholar, the web of science, and the Cochrane database to identify all potential studies for inclusion from January 2000 to January 2020. Conference proceedings, clinicaltrials.gov, reference lists of published trials, reviews, and meta-analyses were also searched. Other authors contributed to sorting out the disagreement to select final eligible studies. Keyword and medical subject heading (MSH) search terms “Atrial fibrillation ablation, Magnetic Resonance Imaging, and atrial fibrosis,” “Ablation and MRI atrial fibrosis,” “atrial fibrosis,” “Late gadolinium enhancement MRI” “LGE MRI,” “DE MRI” “ablation outcomes” were used in several combinations.

After removing the duplicates, we screened a total of 630 studies and selected 78 studies for abstract with or without a full study review. Among the 78 studies, after the abstract review, we sorted 14 studies for full-text review. Finally, we included nine studies in the final analysis.

Following standard criteria were set with the consensus of all authors to finalize the eligible study: 1) Observational study looking into atrial fibrosis *via* LGE-MRI pre-ablation in AF patients, 2) main outcome of interest included recurrence of atrial fibrillation post-ablation, and 3) secondary outcomes included AF recurrence classified based on the degree of fibrosis.

### 2.2 Classification of Fibrosis

To maintain a consistent classification across all the studies, we chose Utah staging to represent the fibrosis stage. Seven of the nine studies selected for this analysis used Utah stages, while two did not. In those that used Utah staging, different cut-off values were used to classify the stages. In the studies by Akoum *et al.* and Mahnkof *et al.*, the Utah stages were classified as Utah I: <5% fibrosis, Utah II: 5–20% fibrosis, Utah III: 20–35% fibrosis, and Utah IV: >35% fibrosis. Similarly, in the studies by Marrouche *et al.*, Luetkens *et al.*, McGann *et al.*, Sramko *et al.*, and Chelu *et al.*, stages were classified as Utah I: <10% fibrosis, Utah II: 10–20% fibrosis, Utah III: 20–30% fibrosis, and Utah IV: >30% fibrosis. Our study used the corresponding stages provided in these seven studies. Oakes *et al.* did not use Utah staging and classified fibrosis stage into three types: mild, moderate, and extensive, representing less than 15%, 15% - 35%, and greater than 35% fibrosis of left atrium, respectively. Similarly, Khurram *et al.* classified the fibrosis stage into less than 35% and greater than 35% fibrosis of the left atrium. For Oakes *et al.*, we categorized mild as Utah I, medium as Utah II, and extensive as Utah IV stages. Due to the limited information to deduce stages I, II, and III in Khurram *et al.*, we lumped the fibrosis to less than 35% as Utah I, II, and III combined and fibrosis greater than 35% as Utah IV stage.

### 2.3 Statistical Analysis

Previous literature indicated that the latter Utah stages (III and IV) were associated with a higher prevalence of AF post-catheter ablation than the earlier stages (I *vs*. II). Thus, the first set of analyses compared stage IV *vs*. stage I (reference group) with seven different studies included. The second set examined the combined stages III and IV *vs*. stages I and II (reference group), with eight studies included. And the third set of analyses compared stage IV *vs*. combined stages I, II, and III (reference group) with seven studies included. In primary studies where no event occurred in one study arm, we added 0.5 to both study arms per the recommendations of Sweeting *et al.* Studies that had no events in both arms were excluded [16]. The metanalysis relied on a random-effects model to pool the odds ratios and 95% confidence intervals using the DerSimonian and Laird method [17]. The data was analyzed using StatsDirect software in England [18].

### 2.4 Ethical Approval

As this paper is a metanalysis, the data used is available from open source. Therefore, our study did not meet the criteria for human subjects, and the institutional review board protocol was waived.

## 3 RESULTS

These studies included articles published from 2009 to 2019. A total of 1,787 patients underwent LGE-MRI before AF ablation. The average age of patients involved in these studies ranged from 59 years to 65 years. The follow-up period was nine months to five years. The baseline characteristics with comorbidities of individual studies are shown in Table **1**. Table **2** displays the number of patients in the individual studies in various stages of atrial fibrosis and AF recurrence based on the stages. The ablation and LGE-MRI process are presented in supplementrary Table **S1**, as reported by the authors in the individual studies.

Fig. (**2**) shows the forest plots displaying the odds ratio (OR) of AF recurrence post-ablation, comparing Utah stage IV fibrosis to Utah Stage I fibrosis. The pooled OR is 9.54 (95% CI, 3.81-28.89) with a *p* < 0.0001. Fig. (**3**) also displays a forest plot that shows the individual and pooled OR of combined stages IV and III to combined stages II and me. The pooled OR from our metanalysis is 2.37 (95% CI, 1.61-3.50), with a *p* < 0.0001. Similarly, Fig. (**4**) shows a forest plot of the individual and pooled OR of stage IV to combined stages III, II, and I. The pooled ratio from our metanalysis is 4.24 (95% CI, 2.39- 7.52, *p* < 0.001).

The combinability of studies was assessed using the I^2^ heterogeneity statistic. The I^2^ statistic was 63% (95% CI, 0% to 81.8%) for the Utah stage I *vs*. IV analysis, 61% (95% CI, 0% to 80.4%) for stage I and II *vs*. III and IV, and 56.8% (95% CI, 0% to 79.5%) for stage I, II & III *vs.* stage IV analysis. Though all three sets of metanalysis had acceptable heterogeneity values, random effects metanalysis was used to ensure robust estimates.

Bias assessment was conducted using a visual funnel plot and statistical tests. A visual inspection of bias using the funnel plots (Figs. **S1**-**S3**) shows some deviation from symmetry. However, all statistical tests for bias were statistically nonsignificant, suggesting the absence of bias.

## 4 DISCUSSION

Our review is the first meta-analysis to investigate the prediction of AF recurrence based on LGE MRI atrial fibrosis to the best of the author's knowledge. This meta-analysis analyzed comprehensive data from 9 studies (n=1,787) evaluating AF recurrence after ablation based on atrial fibrosis by LGE-MRI assessment. The study showed that patients in stage IV are associated with recurrence of AF by 9.54 times after ablation compared with those in stage I. Stages III and IV groups had 2.37 times higher recurrence of AF after ablation compared to stages I and II. The stage IV group had 4.24 times higher recurrence of AF after ablation than the stages I, II, and III groups. Our study has provided compelling evidence of a higher recurrence rate of AF based on the severity of atrial fibrosis detected in LGE-MRI.

AF is known to result from the electrical and structural remodeling of the left atrium [19-21]. But the amount of the total scar burden, the residual fibrosis, and the complete encirclement of pulmonary veins are all predictors of AF recurrences post-ablation [22]. Left atrial chamber dilation and left atrial fibrosis are reported structural remodeling processes [19]. The degree of atrial fibrosis seen in MRI is classified based on the amount of fibrotic atrial tissue. There have been different cut-off values used to classify in various studies. Regardless of the type of classification, these studies have shown an increased recurrence of AF with a higher amount of fibrosis. One can explain this finding by the increasing evidence demonstrating an association between the initiation and perpetuation of AF and increased fibrosis [7]. Recent studies have shown that LGE in MRI has successfully estimated the amount of atrial fibrosis [8, 9]. Various studies in our metanalysis demonstrated increased odds of recurrence of AF post ablation with a higher degree of fibrosis [7-15]. The study by Sramko *et al.* did not find a correlation between atrial fibrosis seen in gadolinium enhancement MRI and the recurrence of AF [10]. The sample size, clinical endpoints, and LGE quantification in the study by Sramko *et al.* were similar to Oakes *et al.* [8]. However, Oakes *et al.* reported a higher recurrence of AF post-ablation in the extensive contrast enhancement group. All of these studies, except the DECAFF study by Marrouche *et al.*, are single-center studies [7]. The DECAFF study is a multicenter study that looked at 15 different centers in the United States, Europe, and Australia and followed 272 patients for 325 and 475 days after a 90-day blanking period and found an increased association between AF recurrence and the degree of fibrosis after adjustments of the confounding variables. Akoum *et al.* followed 120 patients post-ablation ﻿for 283 ± 167 days and found no recurrence in stage Utah stage 1 and 56.3% in the Utah stage 4 group [11]. Similarly, Chelu *et al.* followed 308 patients who underwent AF ablation for five years. They demonstrated an increased hazard ratio of AF recurrence in Utah Stage IV fibrosis compared to stage I [12]. Further, they also observed that when they considered LA fibrosis as a continuous variable, there was a 45% increased risk of recurrence for every 10% increase in fibrosis [12]. The study conducted by Khurram *et al.* observed similar findings when they followed 165 patients post-ablation and concluded an increased recurrence of AF in patients with > 35% of left atrial fibrosis [15]. In another study, Leutkens *et al.* followed 61 patients for 12 months and observed an increased hazard ratio of combined stage III and stage IV compared to stage II and stage I [13]. Besides, Mahnkopf *et al.* and McGann *et al.* followed 333 patients for ﻿324 ± 234 days and ﻿386 patients for one year and concluded that patients with a higher degree of fibrosis have a higher recurrence rate of AF [9, 14]. All these studies have adjusted their results to covariates that might affect the recurrence, such as hypertension, congestive heart failure, mitral valve disease, diabetes, type of AF, left atrial volume, and left ventricular ejection fraction.

Our study, which evaluated the recurrence of AF post-ablation in different atrial fibrosis stages estimated using LGE- MRI, can accurately predict AF post-ablation recurrence with higher odds of recurrence. These findings suggest that the left atrium's structural remodeling significantly affects AF's success, and maybe early ablation before structural remodeling is progressive AF, which is beneficial [23]. This study's results play an essential role in clinical decision-making for physicians and patients in the management of refractory AF.

## 5 LIMITATIONS

There are several limitations to this study. First, all the studies included are observational and lack randomized control trials. Second, all the studies except Marrouche *et al.* are single-center studies. Third, six out of nine studies included are conducted by the CARMA center group (Comprehensive Arrhythmia Research and Management) from Utah; this could limit the studies' generalization. The CARMA center's more extensive studies included a few of the same patient population in the smaller studies. Fourth, error while interpolating the absolute numbers for the recurrence population from the Kaplan-Meier curves in a few studies. Lastly, the expected heterogeneity in classifying fibrosis stages in different studies has been addressed.

## CONCLUSION

This metanalysis demonstrates the strong association between left atrial fibrosis in LGE-MRI and AF post-ablation recurrence. This study will further help physicians quantify the amount of fibrosis and tailor the decisions based on the amount of fibrosis for further management. Further multicenter studies and randomized control trials are still needed to strengthen this finding.

## Figures and Tables

**Fig. (2):**
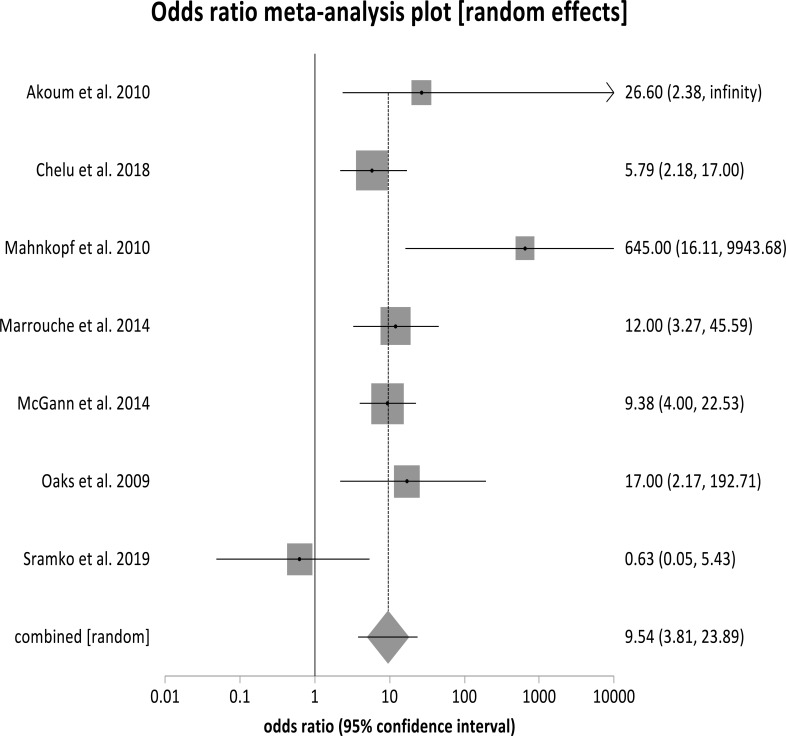
Forest plot showing odds ratio for stage IV to stage I: patients in stage 4 are 9.54 times more likely to have atrial fibrillation recurrence after ablation compared with those in stage 1, pooled OR=9.54 (3.81-28.89), *p* <0.0001.

**Fig. (3):**
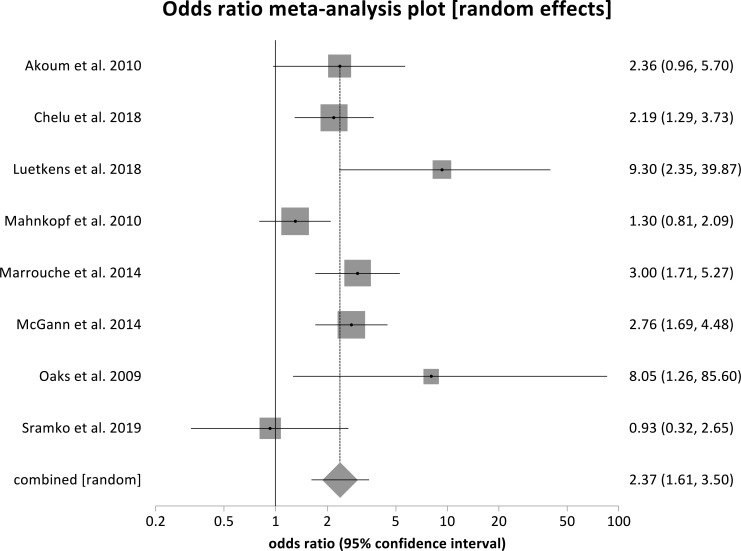
Forest plot showing odds ratios for stages III & IV to stages I & II. Patients in stages 3 & 4 are 2.37 times more likely to have atrial fibrillation recurrence after ablation compared with those in stages 1 & 2, pooled OR = 2.37 (1.61- 3.50), *p* <0.0001.

**Fig. (4):**
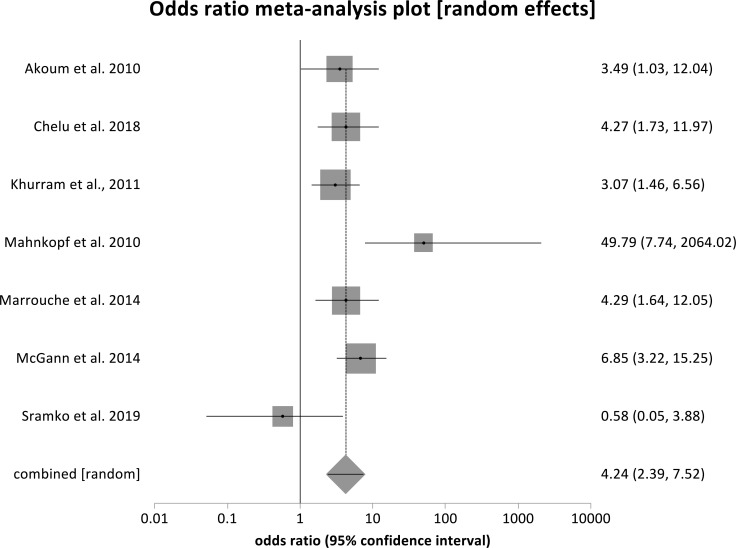
Forest plot showing odds ratios for stage IV to combined staged I, II, &. Patients in stages IV are 4.24 times more likely to have atrial fibrillation recurrence after ablation compared with those in stages I, II, & III, pooled OR = 4.24 (2.39,7.52), *p* <0.001.

**Table 1:** Showing baseline characteristics of the studies included.

**Author**	**Age**	**Female (%)**	**HTN (%)**	**DM (%)**	**CAD (%)**	**CHF (%)**	**CVA/TIA(%)**	**LVEF%**	**Atrial Volume (ml)**	**AAD (%)**	**Paroxysmal (%)**
Akoum *et al.*	63.75 ± 12	N/A	52	10	15%	6.67	N/A	50.7 ± 6.15	N/A	N/A	41
Chelu *et al.*	64.5 ± 12.1	36.6	67	15	N/A	13	22	57.05 ± 10.5	N/A	N/A	N/A
Khurram *et al.*	60.0 ± 10.2	44	46.1	6.7	12.1	11.5	6.1	57.3 ± 6.8	154.8 ± 50.4	N/A	56
Luetkens *et al.*	59.85 ±12.75	34.4	N/A	7.30	N/A	N/A	N/A	N/A	N/A	N/A	65.60
Mahnkopf *et al.*	58.45 ± 8.3	36	57	13.80	14	9	7.80	N/A	N/A	N/A	N/A
Marrouche *et al.*	59 ± 10.7	31.5	55	12.30	10	5.80	5.00	N/A	N/A	63.9	64.60
McGann *et al.*	65 ± 12	36	62	15.20	16.00	9.80	8.80	58 ± 11	51 ± 20	N/A	N/A
Oaks *et al.*	63.6 ± 12.0	35.80	51.9	12.3	11.10	N/A	N/A	52.3 ± 9.8	94.3 ± 41.3	27.2	N/A
Sramko *et al.*	59.5 ± 7	17.50	N/A	N/A	N/A	N/A	N/A	54 ± 5.4	97 ± 34	40	45

**Table 2:** Showing Number of Patients with Fibrosis based on Utah stages and atrial fibrillation recurrence.

**Author**	**Sample Size (N)**	**Total Patients**				**A-fib Recurrence**			
**-**	**-**	**Stage 1**	**Stage 2**	**Stage 3**	**Stage 4**	**Stage 1**	**Stage 2**	**Stage 3**	**Stage 4**
Akoum *et al.*	120	10	71	23	16	0	20	8	9
Chelu *et al.*	308	105	108	61	34	42	54	34	27
Khurram et al.*	165	86	79	19	44	-	-	-	-
Luetkens *et al.*	61	36		25		5		15	
Mahnkopf *et al.*	333	21	141	148	23	0	52	43	22
Marrouche *et al.*	260	49	107	80	24	7	32	36	16
McGann *et al.*	386	133	140	71	42	28	40	24	30
Oaks et al.*	81	40	30	N/A	8	6	13	N/A	6
Sramko *et al.*	73	18	24	24	7	7	10	10	2

**Note:** *Indicates the studies that did not use the UTAH stages of fibrosis for classification.

## LIST OF ABBREVIATIONS

F: Female
HTN: Hypertension
DM: Diabetes Mellitus
CHF: Congestive Heart Failure
CVA/TIA: Cerebrovascular Accident/Transient Ischemic Attack
LVEF: Left Ventricular Ejection Fraction
AV: Atrial Volume
AAD: Antiarrhythmic Drugs
PAF: Paroxysmal Atrial Fibrillation
N/A: Not Available

## SUPPLEMENTARY MATERIAL

PRISMA checklist is available as supplementary material on the publisher’s website along with the published article.
