# Supplementary material for: Prediction of Recurrence of Atrial Fibrillation Post-ablation Based on Atrial Fibrosis Seen on Late Gadolinium Enhancement MRI: A Meta-analysis
Source: Curr Cardiol Rev. 2023 Mar 22;19(3):E051222211571. doi: 10.2174/1573403X19666221205100148 (PMC10280994; doi:10.2174/1573403X19666221205100148)
Supplement: Supplementary file 1 [file CCR-19-E051222211571_SD1.pdf]

Supplementary Material

Prediction of Recurrence of Atrial Fibrillation Post-ablation Based on Atrial Fibrosis Seen on Late Gadolinium Enhancement MRI: A Meta-analysis

Manjari Rani Regmi[1,*], Mukul Bhattarai, Priyanka Parajuli[2], Albert Botchway[3], Nitin Tandan[4], Jumana Abdelkarim[5] and Mohamed Labedi[1]

[1]Division of Cardiology, Southern Illinois University School of Medicine, Springfield, Ill, USA; [2]Columbia University Division of Cardiology at Mount Sinai Medical Center, Miami Beach, FL, USA; [3]Department of Internal Medicine, Southern Illinois University School of Medicine, Springfield, Ill, USA; [4]Division of Cardiology, Ochsner Clinic Foundation, New Orleans, LA, USA; [5]Division of Endocrinology, Southern Illinois University School of Medicine, Springfield, Ill, USA

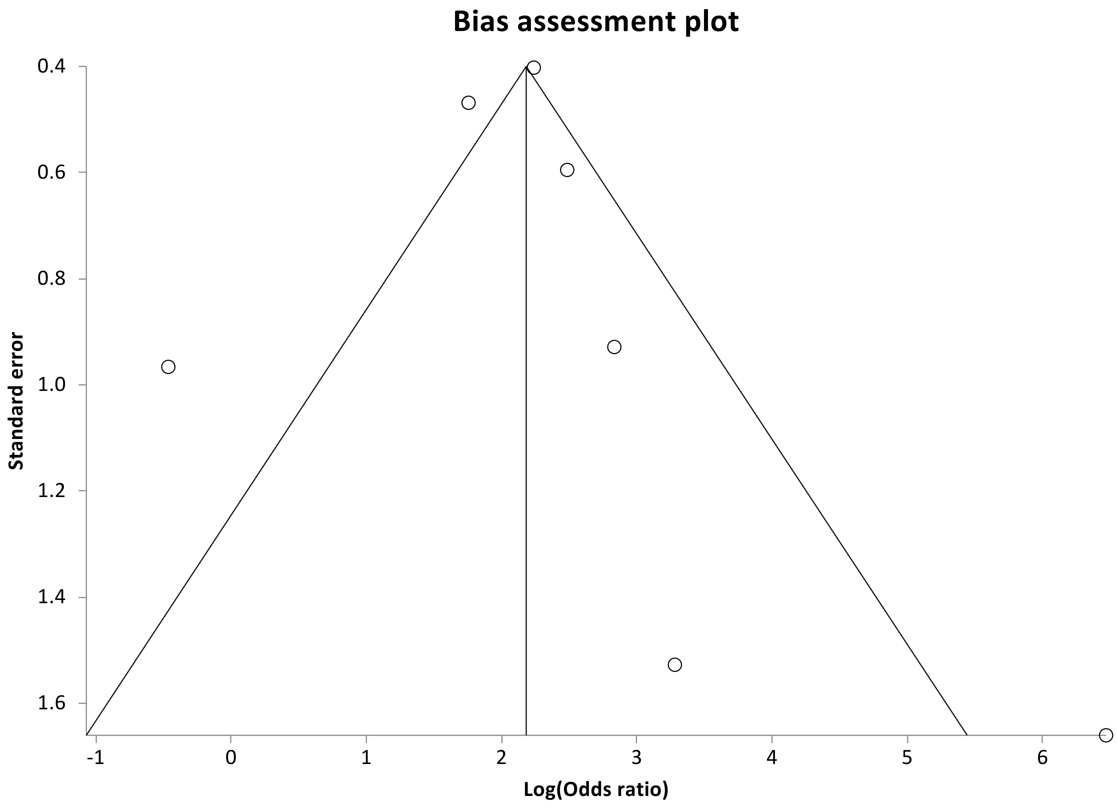

Figure S1. Funnel plot for atrial fibrillation after ablation for **stages I vs. IV**. Egger bias = 1.08 (95% CI -2.70-4.85) P=0.496; Harbord-Egger bias = -0.05 (92.5% CI -4.97-4.87) P=0.982.

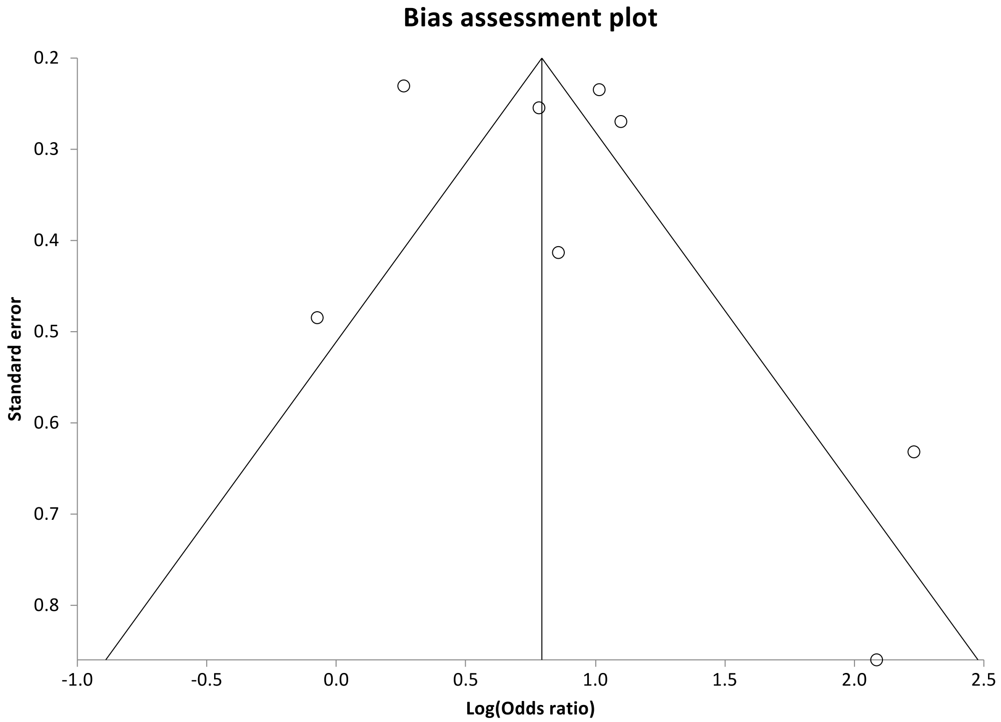

Figure S2. Funnel plot for atrial fibrillation after ablation for **stages I & II vs. III & IV**. Egger bias = 1.64 (95% CI -2.02-5.30) P=0.316; Harbord-Egger bias = 1.80 (92.5% CI -1.67-5.28) P=0.307.

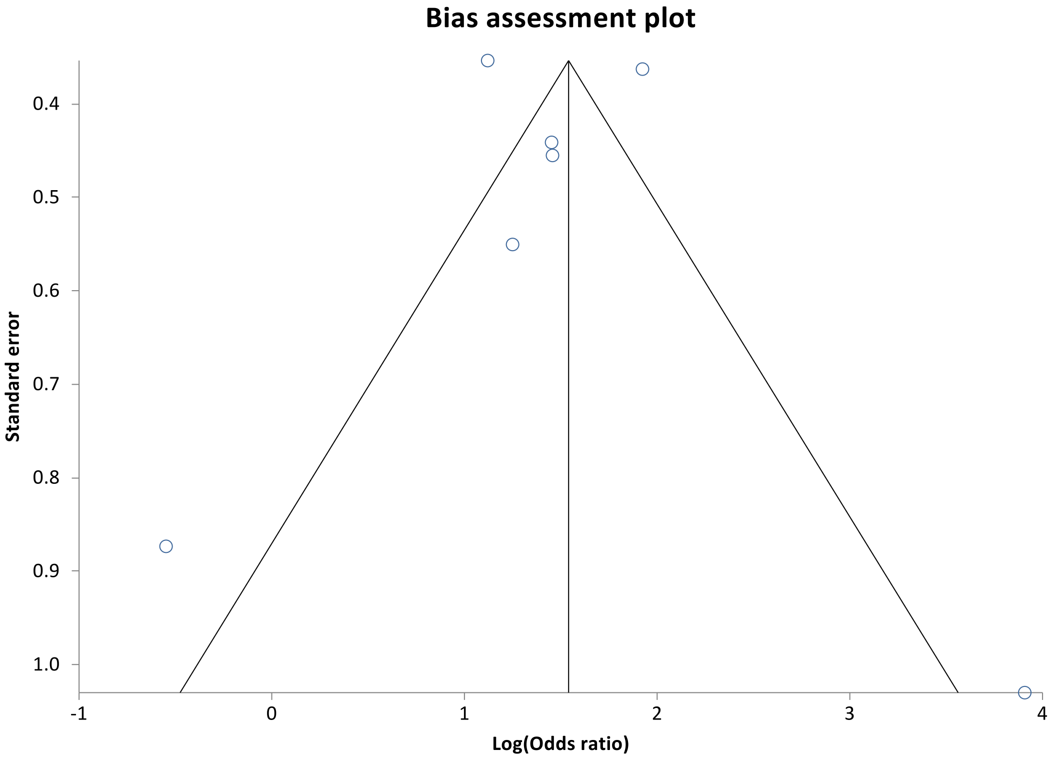

Figure S3. Funnel plot for atrial fibrillation after ablation for **stages I, II& III vs. IV**. Egger bias = 0.15 (95% CI -4.86 – 5.16) P=0.942; Harbord-Egger bias = -2.13 (92.5% CI - 8.45 to 4.18) P=0.483.

Table S1. Table showing methods of ablation and magnetic resonance imaging in all the studies.

Year	Author	Magnetic Resonance Imaging protocol	Ablation Techniques
**2011**	Akoum et al.	All studies were obtained on a 1.5 Tesla Avanto clinical scanner (Siemens Medical Solutions, Erlangen, Germany) using a TIM phased-array receiver coil. The scan was acquired 15 minutes following contrast agent injection (0.1 mmol/kg, Multihance [Bracco Diagnostic Inc., Princeton, NJ]) using a 3D inversion recovery, respiration navigated, ECG- gated, gradient echo pulse sequence. Typical acquisition parameters were: free-breathing using navigator gating, a transverse imaging volume with voxel size = 1.25 × 1.25 × 2.5 mm (reconstructed to 0.625 × 0.625 × 1.25 mm), TR/TE = 5.4/2.3 ms, flip angle = 20°, inversion time (TI) = 270–310 ms, and GRAPPA with R = 2 and 46 reference lines. ECG gating was used to acquire a small subset of phase encoding views during the diastolic phase of the LA cardiac cycle. The time interval between the R-peak of the ECG and the start of data acquisition was defined using the cine images of the LA. Fat saturation was used to suppress fat signal. The TE of the scan (2.3 ms) was chosen such that fat and water are out of phase and the signal intensity of partial volume fat-tissue voxels was reduced allowing improved delineation of the LA wall boundary. The TI value for the DE-MRI scan was identified using a scout scan. Typical scan time for the DE-MRI study was 5–10 minutes depending on subject respiratory and heart rate.	The LA was accessed through 2 transseptal punctures under intracardiac echo guidance using a phased array catheter (Acunav, Siemens Medical Solutions USA, Inc, Mountain View, CA, USA). A 10-pole circular mapping catheter (Lasso, Biosense Webster, Diamond Bar, CA, USA) and a 3.5 mm Thermocool ablation catheter (Biosense Webster) were advanced into the LA for mapping and ablation. A 14- pole catheter (TZ Medical, Portland, OR, USA) was used to record right atrial and coronary sinus electrograms and was used as the reference catheter for 3D electroanatomical mapping with CARTO (Biosense Webster). Radiofrequency energy was delivered with 50 Watts at a catheter tip temperature of 50 °C for no longer than 10 seconds seconds, guided by electrograms abolition recorded on the Lasso catheter. Ablation lesions were placed in a circular fashion in the PV antral region until electrical isolation of the PVs was achieved. Additional lesions were placed along the left atrial posterior wall and septum.
**2018**	Chelu et al.	Images were acquired with either a 1.5-T Avanto or a 3-T Verio clinical MRI scanner (Siemens Healthcare, Erlangen, Germany) using body and spine phased- array receiver coils. Scans were performed %15 minutes after contrast agent injection (0.1 mmol/kg of Multihance; Bracco Diagnostic Inc, Princeton, NJ) using a 3-dimensional inversion recovery prepared, respiration-navigated, ECG-gated, gradient echo pulse sequence. The acquisition parameters were as follows: free breathing using navigator gating, a transverse imaging volume with a voxel size of 1.2591.2592.5 mm (reconstructed to 0.62590.62591.25 mm), inversion time=270 to 320 ms, and generalized autocalibrating partially parallel acquisition with reduction factor R=2. The other scan parameters for LGE-MRI at 1.5-T scanner were as follows: repetition time=5.2 ms, echo time=2.4 ms, and flip angle=20°. Scan parameters for LGE-MRI at 3-T scanner were as follows: repetition time/echo time=3.1/1.4 ms and flip angle=14°. Fat saturation was used to suppress fat signal, and an echo time of the LGE scan was chosen such that fat and water signals were approximately out of phase, resulting in a reduced signal intensity of partial volume fat-tissue voxels and improved delineation of the LA wall boundary. The inversion time was identified using an inversion time scout scan. ECG gating was used to acquire a small subset of phase- encoding views during the diastolic phase of the LA cardiac cycle. The time interval between the R peak of the ECG and the start of data acquisition was defined using cine images of the LA	A 14-pole catheter (TZMedical [Portland, OR] or Bard EP [Lowell, MA]) was used to record right atrial and coronary sinus electrograms and as the reference catheter for 3-dimensional electroanatomical mapping with CARTO 3 (Biosense-Webster, Inc, Diamond Bar, CA). Two transseptal punctures were performed under intracardiac echocardiography guidance using a phased-array catheter (AccuNav; Siemens Medical Solutions USA, Inc, Mountain View, CA). A circular mapping catheter (Lasso; Biosense Webster Inc) and a radiofrequency ablation catheter (Thermo- Cool NaviStar; Biosense Webster, Inc) were advanced into the left atrium for mapping and ablation. Radiofrequency energy was delivered with 50 W (30 mL/min open irrigation) at a catheter tip temperature of 50°C for a maximum duration of 15 seconds and was guided by electrogram abolition. Ablation lesions were placed in a circular manner in the pulmonary vein (PV) antral region until electrical isolation of the PVs was achieved. PV isolation (PVI) was successful in all patients acutely. Radiofrequency delivery was interrupted if the impedance increased suddenly or if a burst in microbubble density was seen by intracardiac echocardiography. Addi- tional LA posterior wall ablation was performed as previously described.
**2016**	Khurram et al.	Images were acquired using a 1.5 Tesla CMR scanner	A double trans-atrial septal puncture was performed under fluoro-

		(Avanto, Siemens, Erlangen, Germany) with a phased array cardiac coil. Contrast enhanced 3D fast low angle shot magnetic resonance angiography images were used to define LA and PV anatomy. LGE- CMR scans were acquired approximately 20 minutes following 0.2 mmol/kg gadolinium injection. The LGE sequence was a 3D inversion recovery prepared respiratory triggered and navigated, ECG gated, and fat suppressed fast spoiled gradient echo sequence (repetition time of 2.5-5.5 ms, echo time of 1.52 ms, field of view at 340 mm, flip angle at 10 degrees, inversion time 240-300 ms, 1.3 × 1.3 mm in-plane spatial resolution, 2 mm slice thickness).	scopic guidance. An endocardial map of the left atrium was created with an electroanatomic mapping system (CARTO, Biosense-Webster) and superimposed upon the pre-existing CMR image of the chamber. With routine hemodynamic and electrocardiographic monitoring, a four-millimeter-tipped irrigated ablation catheter (Thermocool, Biosense-Webster, Diamond Bar, CA) was advanced under fluoroscopic guidance to the left atrium. Circumferential lesions were applied surrounding the pulmonary veins. Additional ostial lesions were targeted to remaining pulmonary vein potentials using a circular multipolar electrode-mapping catheter (Lasso, Biosense, Diamond Bar, CA, USA). Entrance block into the pulmonary veins was confirmed in all patients as the primary procedural endpoint. Additionally, when possible by demonstration of PV capture, exit block was documented. To prevent short-term recurrences of AF, previously ineffective anti-arrhythmic medications were continued for at least 3 months
**2018**	Luetkens et al.	CMR scans were performed on a 1.5 Tesla CMR system (Ingenia 1.5 T, Philips Healthcare, Best, The Netherlands). LA native T1 mapping was performed in end-systole in transversal orientation using a high-resolution 3(3)3(3)5 modified Look-Locker inversion recovery (MOLLI) acquisition scheme18 (acquisition matrix: 320 × 320 mm; time of repetition (TE): 2.18 ms; time of echo (TE): 1.02 ms; parallel imaging factor (SENSE): 2; voxel size (acquired): 2.00 × 2.00 × 8 mm; voxel size (reconstructed): 1.17 × 1.17 × 8 mm; flip angle: 35°; estimated scan duration/breath-hold: 00:15 min). For atrial (LGE) imaging a high-resolution ECG-triggered and navigator gated 3D inversion recovery was performed in transversal orientation covering the entire left atrium. Sequence parameters were as follows: acquisition matrix: 300 × 240 mm; time of repetition (TE): 3.6 ms; time of echo (TE): 1.8 ms; voxel size (acquired): 1.3 × 1.3 × 5.0 mm; voxel size (recon- structed): 0.74 × 0.74 × 2.5 mm; flip angle: 15°; estimated scan duration: 01:54 min. LGE images were acquired 15 minutes after injection of a bolus of 0.2 mmol/kg of body weight of gadobutrol (Gadovist, Bayer Healthcare, Leverkusen, Germany). Optimal inversion time was determined by using the Look-Locker technique	Venous access for PVI was obtained through the right femoral vein. Having positioned a decapolar catheter over a 7 F sheath in the coronary sinus, next a single transseptal puncture (Brockenbrough technique) was performed under fluoroscopic guidance. For selective angiography of the individual pulmonary veins a 15 F sheath (Flexcath ®, Medtronic, Inc., Minneapolis, Minnesota, USA) was used. After angiography, a 28 mm second generation cryoballoon (Arctic Front Advance®; Medtronic, Inc., Minneapolis, Minnesota, USA) was positioned in front of the PV ostium with best possible occlusion, controlled by fluoroscopy. Cryoenergy was applied twice for a period of four minutes each. Prior to isolation of the right superior PV (RSPV), the decapolar catheter was removed from the coronary sinus and positioned in the superior vena cava for continuous stimulation of the phrenic nerve during application of cryoenergy. Recognition of diminishing movements of the diaphragm during fluoroscopy led to instantaneous termination of cry application in the RSPV. Ablation
**2010**	Mahnkopf et al	MRI studies were performed on a 1.5-T Avanto clinical scanner (Siemens Medical Solutions, Erlangen, Germany) using a TIM (Total Imaging Matrix) phased-array receiver coil. The scan was acquired 15 minutes after contrast agent injection (0.1 mmol/kg, Mulithance [Bracco Diagnostic Inc., Princeton, NJ]) using a 3-dimensional inversion recovery, respiration navigated, electrocardiogram (ECG)-gated, gradient echo pulse sequence. Typical acquisition parameters were: free breathing using navigator gating, a transverse imaging volume with voxel size 1.25 × 1.25 × 2.5 mm (reconstructed to 0.625 × 0.625 × 1.25 mm), repetition time/TE = 5.4/2.3 ms, inversion time (TI) = 270 to 310 ms; GRAPPA (Generalized Autocalibrating Partially Parallel Acquisition) with R = 2 and 46 reference lines. ECG gating was used to acquire a small subset of phase-encoding views during the diastolic phase of the LA cardiac cycle. The time interval between the R-peak of the ECG and the start of data acquisition was defined using the cine images of the LA. Fat saturation was used to suppress fat signal. The	A 10-F, 64-element, phased-array ultrasound catheter (Acu-Nav, Siemens Medical Solutions USA, Malvern, PA) was used to visualize the interatrial septum and to guide the transseptal puncture. A circular mapping catheter (Lasso, Biosense Webster, Diamond Bar, CA) and an ablation catheter were inserted into the LA. Intracardiac echocardiography was used to define the pulmonary vein ostia, their antra, and the posterior wall, and was also used to position the circular mapping catheter and ablation catheter. All study patients underwent pulmonary vein antrum isolation (PVAI), defined as electric disconnection of the pulmonary vein antrum from the LA, together with posterior wall and septal debulking.

		TE of the scan (2.3 ms) was chosen such that fat and water are out of phase and the signal intensity of partial volume fat-tissue voxels was reduced, allowing improved delineation of the LA wall boundary. The TI value for the DE-MRI scan was identified using a scout scan. Typical scan time for the DE-MRI study was 5 to 10 minutes depending on subject respiration and heart rate	
**2014**	Marrouche et al.	Customized pulse sequences and imaging protocol for the atrial MRI (Marrek Inc) were installed on 18 Siemens MRI scanners (Siemens Health- care). Ninecentersused 1.5-Tesla scanners,5centersused3-Tesla scanners, and 1 center used both 1.5- and 3-Tesla scanners.	The patients underwent ablation per their institutional protocols. Of the final cohort, 16 patients underwent cryoballoon ablation (6.2%), and the remainder underwent radiofrequency ablation. There were 177 patients (68.1%) who underwent pulmonary vein isolation alone. Forty-three patients (16.5%) underwent cavotricuspid isthmus and pulmonary vein isolation ablation.
**2014**	McGann et al.	High-resolution LGE images of LA were acquired ≈15 minutes after injection of 0.1 mmol/kgmol/kg gadolinium contrast (Multihance, Bracco Diagnostics Inc, Princeton, NJ) using a 3D respiratory-navigated, inversion recovery– prepared GRE pulse sequence with specific parameters published previously.27–29 . Briefly, for this 3D respiratory-navigated, ECG-gated, inversion recovery–prepared GRE pulse sequence, an inversion preparation was applied every heartbeat, and fat saturation was applied immediately before data acquisition. The voxel size is 1.25×1.25×2.5 mm on both 1.5-T and 3-T scanners. Scanning was performed on a 1.5-T Avanto (286 patients) or a 3-T Verio (100 patients) MR scanner (Siemens Healthcare, Erlangen, Germany	A 10-pole circular mapping Lasso catheter and a 3.5-mm Thermocool ablation catheter (Biosense Webster, Diamond Bar, CA) were used, and radiofrequency energy was delivered with 50 W at a catheter tip temperature of 50°C for 5 seconds, guided by electrogram abolition recordings
**2009**	Oaks et al.	All patients underwent MRI studies on a 1.5-T Avanto clinical scanner (Siemens Medical Solutions, Erlangen, Germany) using a TIM phased-array receiver coil or 32-channel cardiac coil (In Vivo Corp, Gainesville, Fla). DE-MRI was acquired approximately 15 minutes after the contrast agent injection (dose, 0.1 mmol/kg body weight; Multihance, Braco Diagnostic Inc, Princeton, NJ) using 3-dimensional (3D) inversion-recovery-prepared, respiration-navigated, ECG- gated, gradient-echo pulse sequence with fat saturation. Typical acquisition parameters were as follows: free breathing using navigator gating, a transverse imaging volume with true voxel size of 1.2 X 1.2 X 2.5 mm, flip angle of 22°, repetition time/echo time of 6.1/2.4 ms, inversion time of 230 to 320 ms, and parallel imaging with GRAPPA technique with R= 2 and 42 reference lines. ECG gating was used to acquire a subset of phase-encoding views during the diastolic phase of the LA cardiac cycle. Typical scan time for the DE-MRI study was 5 to 9 minutes, depending on the subject's respiration and heart rate.	Briefly, a 10F, 64- element, phased-array ultrasound catheter (AcuNav, Siemens Medical Solutions USA, Malvern, Pa) was used to visualize the interatrial septum and to guide the transseptal puncture. A circular mapping catheter (Lasso, Biosense Webster) and an ablation catheter were inserted into the LA. Intracardiac echocardiography was used to define the PV ostia, their antra, and the posterior wall. Intracardiac echocardiography also was used to position the circular mapping catheter and ablation catheter.
**2019**	Sramko et al.	The imaging was performed on a 1.5 Tesla Scanner (Avanto, Siemens Medical Solutions, Erlangen, Germany) using a 12-channel body coil. A stack of contiguous short-axis cine images of the entire LA was acquired to assess the LA function (typical temporal resolution of40 ms, voxel size of2.1 × 1.6 × 8mm). Subsequently, time-resolved 3D angiography of the LA (voxel size of 1.7 × 1.7 × 1.7 mm) was obtained with a bolus injection of 0.2 mmol/kg organoboron. Acquisition of the LGE images started 10min after administration of the contrast using a segmented3DFLASHsequencewith FatSat. Typical pa-	The procedure was guided by a 3D mapping system (CARTO, Biosense Webster, Diamond Bar, CA) and intra-cardiac echocardiography. Ablation was performed using a 3.5-mm irrigated-tip catheter (NaviStar Thermocool, Biosense Webster, Diamond Bar, CA). The pulmonary veins were isolated by circumferential point-by-point lesions; additional linear lesions were performed in the patients with persistent AF. Radiofrequency energy of up to 35 W was applied at each point for 30–60 s.

		rameters were: TR/TE of 4.8/1.5 ms, TI of270 ms, 1 inversion pulse per RR, flip angle of10°, linear k-space filling, FOV of 400 × 400 × 100 mm, voxel size of 1.6 × 1.6 × 3 mm interpolated to 0.78 × 0.78× 1.7mm. The images were acquired during free breathing with a navigator (PACE, acceptance window of±2.5 mm at end-expiration). ECG-triggering was adjusted using a 4-chamber view cine loop to obtain a subset of images during a cardiac phase with the least LA motion. The average acquisition time for the LGE-MRI study was 11 ± 4 min.	

**Section and Topic**	**Item #**	**Checklist item**	**Location where item is reported**
**TITLE**			
Title	1	Identify the report as a systematic review.	Title
**ABSTRACT**			
Abstract	2	See the PRISMA 2020 for Abstracts checklist.	
**INTRODUCTION**			
Rationale	3	Describe the rationale for the review in the context of existing knowledge.	Introduction
Objectives	4	Provide an explicit statement of the objective(s) or question(s) the review addresses.	Introduction
**METHODS**			
Eligibility criteria	5	Specify the inclusion and exclusion criteria for the review and how studies were grouped for the syntheses.	Study Selection
Information sources	6	Specify all databases, registers, websites, organisations, reference lists and other sources searched or consulted to identify studies. Specify the date when each source was last searched or consulted.	Study Selection
Search strategy	7	Present the full search strategies for all databases, registers and websites, including any filters and limits used.	Study Selection
Selection process	8	Specify the methods used to decide whether a study met the inclusion criteria of the review, including how many reviewers screened each record and each report retrieved, whether they worked independently, and if applicable, details of automation tools used in the process.	Methods
Data collection process	9	Specify the methods used to collect data from reports, including how many reviewers collected data from each report, whether they worked independently, any processes for obtaining or confirming data from study investigators, and if applicable, details of automation tools used in the process.	Methods
Data items	10a	List and define all outcomes for which data were sought. Specify whether all results that were compatible with each outcome domain in each study were sought (e.g. for all measures, time points, analyses), and if not, the methods used to decide which results to collect.	Methods
	10b	List and define all other variables for which data were sought (e.g. participant and intervention characteristics, funding sources). Describe any assumptions made about any missing or unclear information.	N/A
Study risk of bias assessment	11	Specify the methods used to assess risk of bias in the included studies, including details of the tool(s) used, how many reviewers assessed each study and whether they worked independently, and if applicable, details of automation tools used in the process.	Methods
Effect measures	12	Specify for each outcome the effect measure(s) (e.g. risk ratio, mean difference) used in the synthesis or presentation of results.	Methods
Synthesis methods	13a	Describe the processes used to decide which studies were eligible for each synthesis (e.g. tabulating the study intervention characteristics and comparing against the planned groups for each synthesis (item #5)).	Methods
	13b	Describe any methods required to prepare the data for presentation or synthesis, such as handling of missing summary statistics, or data conversions.	Methods
	13c	Describe any methods used to tabulate or visually display results of individual studies and syntheses.	N/A
	13d	Describe any methods used to synthesize results and provide a rationale for the choice(s). If meta-analysis was performed, describe the model(s), method(s) to identify the presence and extent of statistical heterogeneity, and software package(s) used.	Statistical analyses
	13e	Describe any methods used to explore possible causes of heterogeneity among study results (e.g. subgroup analysis, meta-regression).	Supplement document
	13f	Describe any sensitivity analyses conducted to assess robustness of the synthesized results.	Statistical analyses

Section and Topic	Item #	Checklist item	Location where item is reported
Reporting bias assessment	14	Describe any methods used to assess risk of bias due to missing results in a synthesis (arising from reporting biases).	Supplement document
Certainty assessment	15	Describe any methods used to assess certainty (or confidence) in the body of evidence for an outcome.	Supplement document
**RESULTS**			
Study selection	16a	Describe the results of the search and selection process, from the number of records identified in the search to the number of studies included in the review, ideally using a flow diagram.	Prisma flow diagram
	16b	Cite studies that might appear to meet the inclusion criteria, but which were excluded, and explain why they were excluded.	Prisma flow diagram
Study characteristics	17	Cite each included study and present its characteristics.	Table. 1
Risk of bias in studies	18	Present assessments of risk of bias for each included study.	Supplement document
Results of individual studies	19	For all outcomes, present, for each study: (a) summary statistics for each group (where appropriate) and (b) an effect estimate and its precision (e.g. confidence/credible interval), ideally using structured tables or plots.	Table 2.
Results of syntheses	20a	For each synthesis, briefly summarise the characteristics and risk of bias among contributing studies.	Supplement document
	20b	Present results of all statistical syntheses conducted. If meta-analysis was done, present for each the summary estimate and its precision (e.g. confidence/credible interval) and measures of statistical heterogeneity. If comparing groups, describe the direction of the effect.	Figure 1-4
	20c	Present results of all investigations of possible causes of heterogeneity among study results.	Supplement Document
	20d	Present results of all sensitivity analyses conducted to assess the robustness of the synthesized results.	Figure 1-4
Reporting biases	21	Present assessments of risk of bias due to missing results (arising from reporting biases) for each synthesis assessed.	N/A
Certainty of evidence	22	Present assessments of certainty (or confidence) in the body of evidence for each outcome assessed.	Figure 1-4
**DISCUSSION**			
Discussion	23a	Provide a general interpretation of the results in the context of other evidence.	Discussion
	23b	Discuss any limitations of the evidence included in the review.	Discussion
	23c	Discuss any limitations of the review processes used.	Discussion
	23d	Discuss implications of the results for practice, policy, and future research.	Discussion
**OTHER INFORMATION**			
Registration and protocol	24a	Provide registration information for the review, including register name and registration number, or state that the review was not registered.	N/A
	24b	Indicate where the review protocol can be accessed, or state that a protocol was not prepared.	N/A
	24c	Describe and explain any amendments to information provided at registration or in the protocol.	N/A
Support	25	Describe sources of financial or non-financial support for the review, and the role of the funders or sponsors in the review.	N/A
Competing interests	26	Declare any competing interests of review authors.	Cover letter
Availability of data, code and other materials	27	Report which of the following are publicly available and where they can be found: template data collection forms; data extracted from included studies; data used for all analyses; analytic code; any other materials used in the review.	Methods

From: Page MJ, McKenzie JE, Bossuyt PM, Boutron I, Hoffmann TC, Mulrow CD, et al. The PRISMA 2020 statement: an updated guideline for reporting systematic reviews. BMJ 2021;372:n71. doi: 10.1136/bmj.n71
For more information, visit: http://www.prisma-statement.org/
